# Low Prevalence of HEV Infection and No Associated Risk of HEV Transmission from Mother to Child among Pregnant Women in Vietnam

**DOI:** 10.3390/pathogens10101340

**Published:** 2021-10-17

**Authors:** Pham Xuan Huy, Dang Thanh Chung, Dang Thuy Linh, Ngo Thu Hang, Sivaramakrishna Rachakonda, Srinivas Reddy Pallerla, Le Thi Kieu Linh, Hoang Van Tong, Le Minh Dung, Can Van Mao, Heiner Wedemeyer, C-Thomas Bock, Peter G. Kremsner, Le Huu Song, Bui Tien Sy, Nguyen Linh Toan, Thirumalaisamy P. Velavan

**Affiliations:** 1 Department of Pathophysiology, Vietnam Military Medical University, Hanoi 100000, Vietnam; huyphamhvqy@gmail.com (P.X.H.); dtchungjp@gmail.com (D.T.C.); danglinh46bhvqy@gmail.com (D.T.L.); ngohang_k34a@yahoo.com (N.T.H.); hoangvantong@vmmu.edu.vn (H.V.T.); canvanmao@vmmu.edu.vn (C.V.M.); 2 Institute of Tropical Medicine, Universitätsklinikum Tübingen, 72074 Tübingen, Germany; krishna.rachakonda@gmail.com (S.R.); srinivas-reddy.pallerla@uni-tuebingen.de (S.R.P.); le.linh@klinikum.uni-tuebingen.de (L.T.K.L.); peter.kremsner@uni-tuebingen.de (P.G.K.); 3 Vietnamese-German Center for Medical Research (VG-CARE), Hanoi 100000, Vietnam; lehuusong@108-icid.com (L.H.S.); tiensy2015@yahoo.com (B.T.S.); 4 Tra Vinh Obstetrics and Pediatrics Hospital, Tra Vinh 940000, Vietnam; bsleminhdung67@gmail.com; 5 Department of Gastroenterology, Hepatology and Endocrinology, Hannover Medical School, 30625 Hannover, Germany; Wedemeyer.heiner@mh-hannover.de; 6 German Center for Infection Research, Partner Hannover-Braunschweig, 38124 Braunschweig, Germany; 7 Department of Infectious Diseases, Division of Viral Gastroenteritis and Hepatitis Pathogens and Enteroviruses, Robert Koch Institute, 13353 Berlin, Germany; BockC@rki.de; 8 Centre de Recherches Medicales de Lambarene, Lambaréné B.P. 242, Gabon; 9 108 Military Central Hospital, Hanoi 100000, Vietnam

**Keywords:** hepatitis E virus, HEV-3, pregnant women, mother-to-child-transmission, zoonoses

## Abstract

Infections with HEV in low- and middle-income countries (LMICs) are associated with increased rates of preterm birth, miscarriage, and stillbirth. The aim of the present study was to investigate HEV infections in pregnant women and the possibility of mother-to-child transmission, and associated outcomes. A total of 183 pregnant women in their third trimester were recruited and followed until delivery. Anti-HEV IgG and IgM were determined via enzyme-linked immunosorbent assay (ELISA), and HEV nucleic acids were detected in stool and cord blood samples. HEV genotypes were identified by Sanger sequencing, and phylogenetic analyses were performed. Mother-to-child transmission and associated adverse outcomes were not observed. Only 2% of patients (*n* = 4/183) tested positive for anti-HEV IgM, and 8% (*n* = 14/183) tested positive for anti-HEV IgG antibodies. Cord blood (*n* = 150) analysis showed that there was no IgM detected, while 4% (*n* = 6/150) tested positive for anti-HEV IgG, which was consistent with mothers testing positive for anti-HEV IgG. Nucleic acid tests for HEV RNA yielded 2% (*n* = 4/183) from the serum and stool of pregnant women, and none from cord blood. The HEV isolates belonged to the genotype HEV-3a, with 99% homology with humans and 96% with pigs. No association was found between the risk of HEV infection and pregnancy outcomes or HEV transmission from mother to child. HEV-3 infections of zoonotic origin in pregnancy might have eventually resolved without complications.

## 1. Introduction

The hepatitis E virus (HEV) is a common cause of acute viral hepatitis worldwide, with an estimated 20 million HEV infections, 3.4 million symptomatic infections, 70,000 deaths, and 3000 stillbirths per year [1]. Though the disease is usually a mild and self-limiting form of acute hepatitis, pregnant women in endemic countries are at particular risk of severe disease, as pregnant women infected with HEV genotype 1 (HEV-1) in the third trimester are at high risk, with relative mortality and morbidity of ~15–60% [2]. HEV accounts for nearly 61% of cases from East and South Asia [3,4]. HEV infections are well known, especially the fecal–oral route of transmission in low- and middle-income countries (LMICs), including Vietnam, where seasonal monsoons leading to flooding are common. Major outbreaks of HEV infection were reported from Asia and South-East Asia [1].

The clinical complications of HEV often include acute liver failure, bleeding, and eclampsia gravidarum. The mechanism of predilection in pregnant women is not understood, and attributed causes include fulminant liver failure and obstetric complications such as eclampsia and hemorrhage [5,6,7]. However, it is believed that hormonal (progesterone and estrogen) and existing immune conditions, high viral loads, nutritional status, and host factors may promote fatal HEV-1 courses during pregnancy [2]. Infections with HEV genotype 1 (HEV-1) are the greatest contributor, and are associated with increased rates of preterm birth, miscarriage, and stillbirth [8,9,10,11]. Acute and chronic HEV genotype 3 (HEV-3) infections (zoonotic origin) have also been reported in pregnant women, but the infections eventually resolve without complications.

The incubation period of HEV in acute infection is 2–9 weeks (15–64 days). The diagnosis of HEV is based on serological and molecular methods, with each having distinct advantages [1,12,13]. RNA can usually be detected in the blood 2–6 weeks after infection, and in the stool for ~2 weeks longer. HEV-specific IgM antibodies appear in the bloodstream 3-4 weeks after infection, and persist between 4 and 6 months, while IgG antibodies can be detected for several years after recovery [2]. 

Our previous studies of the Vietnamese population have revealed a high anti-HEV IgG seroprevalence in healthy individuals (31%), in chronic hepatitis B virus (HBV) patients (45%), and among occupational workers exposed to pigs (53%) [4,14,15]. Despite the possibility of such serious outcomes, the scale of HEV infection during pregnancy, and of potential maternal, fetal, and neonatal complications, has not been investigated in resource-poor settings where the prevalence of HEV infection is expected to be high. Furthermore, long-term growth and neurodevelopmental abnormalities in children exposed to HEV in utero represent a critical evidence gap. 

Therefore, in the present study, the incidence of HEV infection was determined by testing anti-HEV IgG/IgM and HEV RNA in pregnant Vietnamese women in their third trimester, and from the corresponding cord blood of the newborn. The data obtained were correlated with clinical outcomes.

## 2. Results

The present study aimed at understanding the seroprevalence of HEV in Vietnamese women during their third trimester of pregnancy, as well as the possibility of mother-to-child-transmission. HEV status was assessed via serological (IgM and IgG antibodies) and PCR-based methods (HEV RNA) in the sera and stool of participants, and was correlated to other risk factors that contribute to the spread of infection. 

### 2.1. Baseline Characteristics of the Study Population

The median age of the cohort was 27 years, with an interquartile range (IQR) of 24-32 years (Table 1). None of the women had any pregnancy-related complications. Out of 183 women, 79% (145) had a full-term gestation, while 21% (38) had premature delivery. Women with first-time pregnancy comprised 49% (89) of the study population, while the remaining 51% (91) had had previous pregnancies. History of abortions and miscarriages were reported in 9% (17) and 14% (26), respectively, while the remaining 91% (164) and 86% (157), respectively, had no such history. None of the women had jaundice during pregnancy, but 1% (2) had postpartum jaundice. The other demographic parameters indicated that 87% (160) lived in rural areas compared to 13% (23) in urban areas, and 91% (168) consumed either bore-well water or tap water compared to 9% (15) rainwater.

### 2.2. Anti-HEV IgM and IgG Seroprevalence in Pregnant Women

Serological tests revealed that 2% (4 of 183) of the women carried anti-HEV IgM antibodies, while the remaining 98% (179) were negative for anti-HEV IgM antibodies. Tests for anti-HEV IgG antibodies showed that 8% (14 of 183) of the pregnant women were positive, while the remaining 92% (169) were negative. In a subset of women comprising 82% (150 of 183) of the cohort, the umbilical cord blood was also screened by serological assays to assess the presence of the virus in neonates. Anti-HEV IgM antibodies were absent in all cord blood samples, but anti-HEV IgG antibodies were detected in 4% (6 of 150). Incidentally, the mothers of the six infants also showed anti-HEV IgG positivity.

### 2.3. Genotype and Phylogenetic Analysis

**Figure 1:**
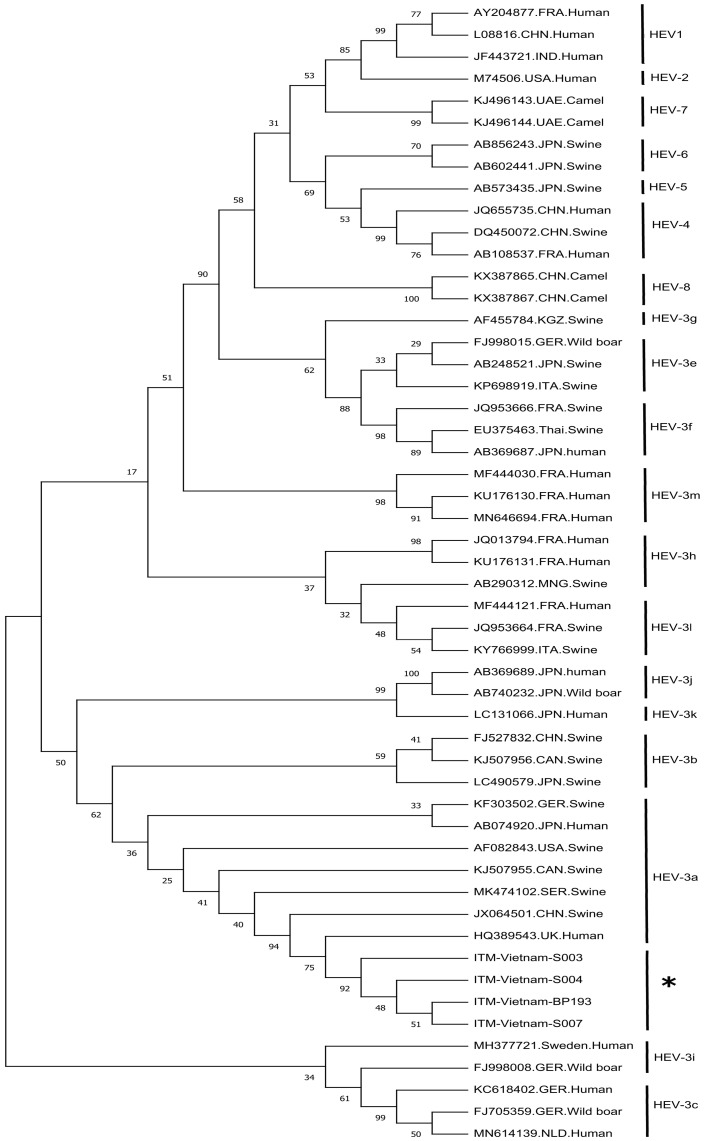
Maximum likelihood phylogenetic tree of HEV nucleotide sequences of ORF1 isolated from humans and animals. The sequences analyzed in the present study are marked with asterisks (*), and the NCBI GenBank accession numbers are OK129292-OK129295.

PCR-based gene sequencing of 306 bp fragments of ORF1 (RNA-dependent RNA polymerase, RdRp) identified HEV RNA in 2% (4 of 183) of the women. Of the four, HEV RNA was detected in three serum samples and one stool sample. HEV RNA was not detected in any of the 4 IgM or 14 anti-HEV IgG serologically positive women. Nucleotide BLAST (BLASTn) analysis of four RNA sequences revealed more than 99% and 96% homology with HEV genotype-3a (HEV-3a) isolates from humans (Kernow-C1 strain) and swine (swCNGX760-10; SRB-32-S/2018), respectively. In addition, a phylogenetic tree of RdRp-ORF1 fragments obtained from different subtypes of HEV confirmed that the four isolates in the present study aligned more closely to HEV genotype 3a (HEV-3a) (Acc: HQ389543, Kernow-C1 strain), compared to other subtypes (Figure 1).

### 2.4. Correlation of Serological and Genomic Results

There was no correlation between serological and molecular tests. All four ELISA anti-HEV-IgM-positive samples were negative for HEV PCR and, similarly, all four PCR-positive samples were negative for anti-HEV IgM antibodies. Combining the results of ELISA (HEV IgM or IgG) and PCR (HEV RNA) broadly showed three groups in the study population—IgM and RNA were found in 4% (*n* = 8), and IgG in 8% (*n* = 14). Comparing the three groups (IgM, RNA, and IgG; HEV-negative) based on clinical or paraclinical parameters did not reveal any differences (Table 2). Liver enzymes and hematological parameters such as AST (aspartate transaminase), ALT (alanine transaminase), and platelet count were in normal ranges in all three groups. The birth weight of the newborns was comparable in all three groups. There were no concurrent infections of hepatitis B or C virus (HBV or HCV) or HIV in any of the women with either IgM/RNA or IgG, but four (2%) women in the HEV-negative group tested positive for HBV (Table 2). Demographic parameters such as rural/urban lifestyle, source of drinking water, previous pregnancies, history of abortions and miscarriages, and history of blood transfusions also did not reveal any differences between the groups (Table 2). 

## 3. Discussion

Many South-East Asian countries, including Vietnam, have a high seroprevalence of anti-HEV antibodies (22–77%), which can be attributed to various factors [16]. In Vietnam—a country where HEV and HBV infections are endemic—pregnant women are at high risk of HEV infection, which is often caused by HEV genotype 1 (HEV-1). However, there is little literature on the seroprevalence of HEV in pregnant women, particularly in Vietnam and South-East Asia, where HEV genotype 3 (HEV-3), followed by HEV genotype 4 (HEV-4), have been reported as the predominant HEV genotypes [17]. Little is known about infections caused by the HEV-3 genotype during pregnancy and their clinical consequences [9,18]. This study adds to previous findings that HEV infections caused by the HEV-3 genotype during pregnancy are typically resolved without complications.

In the present cross-sectional study, involving only 183 pregnant women, we confirmed the presence of the HEV genotype 3a (HEV-3a) in 2% of the studied subjects. Our in silico analysis based on nucleotide BLAST and phylogeny revealed > 96% sequence homology for viral sequences to HEV genotype 3a (HEV-3a) isolates from pigs. This is consistent with our previous study, which also confirmed in another meta-analysis that people who have frequent contact with pigs or pig products have a twofold increased risk of higher anti-HEV seropositivity compared to the general population [4,19].

The combined prevalence of serological and nucleic acid detection methods in our study showed anti-HEV IgM and RNA in 4% (8 of 183) of women. Our previous study in individuals who had not been exposed to pigs/pork showed an anti-HEV IgM rate of 6% in the general population [4]. There is no evidence of elevated liver enzymes (AST and ALT) in people who test positive for both anti-HEV IgM and RNA, or in people who test positive for anti-HEV IgG only, indicating a low HEV viral load and, thus, a self-limiting infection. Moreover, acute liver failure—a severe consequence of HEV infection in pregnant women—is rare in those with HEV-3 or -4 genotypes, and observed mostly in persons with HEV-1 or pre-existing liver diseases [20,21]. HEV genotype 1 (HEV-1) is transmitted via the oral–fecal route, as opposed to zoonotic transmission of HEV genotype 3 (HEV-3) [9,17,22,23,24]. 

Mother-to-child transmission and associated adverse outcomes were not observed. All seropositive women had a successful delivery, and no maternal, prenatal, or neonatal complications were reported. A comparison of the birth weight of the newborns in the groups with and without HEV infection also showed no significant differences. The presence of anti-HEV IgG antibodies in 8% of pregnant women suggests that these individuals had either been previously exposed to HEV infection or were convalescing. [9].

Our results lacked concordance between serological and genomic tests. It is plausible that HEV infection in eight individuals (with IgM/RNA) could be in different phases. PCR, being a sensitive assay, detects viral genomes prior to the manifestation of clinical symptoms in patients. On the other hand, IgM antibodies could only be detected after the onset of infection. Hence, HEV antigens are inversely related to the concentration of HEV IgM antibodies, explaining the lack of correlation between serological and genomic test results [13,25,26]. The ELISA kit used in the study (Wantai Beijing) is very sensitive, with minimal cross-reactivity [26,27].

Possible transplacental transmission of anti- HEV IgG to six neonates was observed. This is consistent with previous reports of instances of IgG transmission to neonates [28,29]. The present study is not devoid of limitations, as the cohort size was small and the number of events recorded was very low. In addition, participants were non-randomized and from a single center. Another limitation of the study was that we did not examine the placentae of pregnant women who were positive for anti-HEV IgM antibody and HEV RNA. Due to the smaller number of events, a relatively larger sample number would be needed for comprehensive investigation of the HEV epidemiology in pregnant women.

## 4. Materials and Methods

### 4.1. Ethics Statement

This study was approved by the Institutional Review Board of Vietnam Military Medical University, Hanoi, Vietnam. Informed written consent was obtained at the time of sampling from all study participants.

### 4.2. Study Design

Between January 2019 and December 2019, a total of 183 pregnant women aged more than 16 years in their third trimester who had consented to participate in the study were recruited and followed until delivery. Recruitment took place at the Department of Obstetrics and Gynecology, Tra Vinh Obstetrics and Pediatrics Hospital, Tra Vinh Province, Vietnam. Cord blood samples from newborns were collected for the study from mothers whose family or legal guardians had given consent. Subjects were carefully screened by obstetricians to collect general information about the patients and factors associated with the risk of HEV infection, such as sociological characteristics, obstetric history, pregnancy outcomes, and neonatal status after birth. Five milliliters of venous blood and stool samples were collected from all participants for serological and genomic analysis. The sera were separated and stored until further use.

### 4.3. Serological Assays

Anti-HEV IgG and IgM were determined in sera from pregnant women via enzyme-linked immunosorbent assays (ELISAs), using Wantai HEV ELISA (Beijing Wantai Biological Pharmacy Enterprise Co., Ltd., Beijing, China), according to the manufacturer’s instructions. The assay was considered positive when the optical density was ≥ 0.4 + nonreactive control means (NRCx) or ≥ 0.5 + NRCx, for IgM and IgG, respectively. The positivity absorbance was measured at 450 nm using a plate reader. The proposed sensitivity and specificity of the IgM assay were 98% and 97%, respectively, while those for IgG were 99.9% and 99%, respectively, and results from the IgM assay were expected to have 67% concordance with the HEV RNA test.

### 4.4. HEV Nucleic Acid Testing from Sera and Stool Samples

Total RNA was extracted from 183 serum and stool samples and 150 cord blood samples using either 140 µl serum or 100 µl stool suspensions with the QIAamp Viral RNA Mini Kit (QIAGEN GmbH, Hilden, Germany). The extracted HEV RNA was reversely transcribed into complementary DNA using the QuantiTect Reverse Transcription Kit (QIAGEN GmbH), and amplified by PCR as previously described [4]. In brief, primers were designed based on the RNA-dependent RNA polymerase (RdRp) region, which belongs to the viral *ORF1*. The outer primer pairs were HEV-38 (sense) 5′-GAG GCY ATG GTS GAG AAR G-3′ and HEV-39 (antisense) 5′-GCC ATG TTC CAG ACR GTR TTC C-3′; the inner primers were HEV-37 (sense) 5′-GGT TCC GYG CTA TTG ARA ARG-3′ and HEV-27 (antisense) 5′-TCR CCA GAG TGY TTC TTC C-3′. Amplified PCR products were purified (Exo-SAP-IT kit; USB, Affymetrix, Santa Clara, CA, USA) and sequenced using an ABI 3130XL sequencer (BigDye Terminator v3.1 cycle sequencing kit; Applied Biosystems, Foster City, CA, USA).

### 4.5. HEV Phylogenetic Analysis

All sequences were edited and aligned using BioEdit software version 7.2 (http://bioedit.software.informer.com/7.2, accessed on 15 March 2021) and the CLUSTAL Muscle algorithm. HEV genotyping was performed via phylogenetic analyses based on sequences of the ORF1 RdRp region using the MEGAX software [30]. The sequences were submitted to the NCBI GenBank database (accession numbers: OK129292-OK129295). Phylogenetic trees were constructed using maximum likelihood (Kimura 2-parameter model). Statistical robustness and reliability of the branching order were confirmed by bootstrap analysis using 1000 reiterations. All HEV reference sequences belonging to the 8 HEV genotypes were obtained from the NCBI GenBank.

## 5. Conclusions

No association was found between the risk of HEV infection and pregnancy outcomes or HEV transmission from mother to child. HEV genotype 3 (HEV-3) infections of zoonotic origin in pregnancy might have eventually resolved without complications. The results and conclusions of the present study warrant further studies with the larger samples that are needed to shed light on this important issue.

## Figures and Tables

**Table 1:** Baseline data of the recruited pregnant women.

**Parameters**	**Pregnant Women;** *n* = 183 (%) or (IQR)
Age (years)	
Median (IQR)	27.9 (24–32)
Accommodation	
Rural	160 (87)
Suburban	23 (13)
Source of drinking water	
Rain	15 (8)
Bore/Tap	168 (92)
Past pregnancies	
None	89 (49)
One or more	91 (49)
Data missing	3 (2)
History of abortions	
None	164 (90)
One or more	17 (9)
Data missing	2 (1)
History of miscarriages	
None	157 (86)
One or more	26 (14)
History of blood transfusion	
No	175 (96)
Yes	8 (4)
History of jaundice	
No	181 (99)
Yes	2 (1)
Hepatitis B virus	
No	179 (98)
Yes	4 (2)
Full-term birth (37–42 weeks)	
No	38 (21)
Yes	145 (79)
Postpartum jaundice	
No	181 (99)
Yes	2 (1)
AST (normal range ≤ 37 U/L)	
Median (IQR)	17.23 (13–20)
ALT (normal range ≤ 40 U/L)	
Median (IQR)	12.54 (9–14)
Platelet count	
Median (IQR)	229 (194–258)

**Table 2:** Baseline data stratified based on PCR and serological tests.

**Parameters**	**anti-HEV-negative and RNA-negative** n = 161 (%)/(IQR)	**anti-HEV-IgG-positive** n = 14 (%)/(IQR)	**anti-HEV-IgM-positive** n = 4 (%)/(IQR)	**HEVRNA-positive** n = 4 (%)/(IQR)
Age (years)	27 (24–32)	29.5 (24–35)	28 (26–29)	27 (24–30)
Median (IQR)				
Accommodation				
Rural	139 (86)	13 (93)	4 (100)	4(100)
Suburban	22 (14)	1 (7)	0 (0)	0 (0)
Source of drinking water				
Rain	14 (9)	1 (7)	0 (0)	0 (0)
Bore/Tap	147 (92)	13 (93)	4 (100)	4 (100)
Past pregnancies				
None	80 (50)	5 (36)	2(50)	2 (50)
One or more	78 (48)	9 (64)	2 (50)	2 (50)
Data missing				
History of abortions				
None	144 (89)	13 (93)	3 (75)	4 (100)
One or more	13 (11)	1 (7)	1 (25)	0 (0)
Data missing				
History of miscarriages				
None	140 (87)	10 (71)	4(100)	3 (75)
One or more	21 (13)	4 (29)	0 (0)	1 (25)
History of blood transfusion				
No	154 (96)	13 (93)	4 (100)	4 (100)
Yes	7 (4)	1 (7)	0 (0)	0 (0)
History of jaundice				
No	160 (99)	13 (93)	4 (100)	4 (100)
Yes	1 (1)	1 (7)	0 (0)	0 (0)
Hepatitis B virus				
No	155 (96)	0 (0)	0 (0)	0 (0)
Yes	4 (3)	0 (0)	0 (0)	0 (0)
Full-term birth (37–42 weeks)				
No	33 (20)	4 (29)	1 (25)	0 (0)
Yes	128 (80)	10 (71)	3 (75)	4 (100)
Postpartum jaundice				
No	159 (99)	14 (100)	4 (100)	4 (100)
Yes	2 (1)	0 (0)	0 (0)	0 (0)
AST (normal range ≤ 37 U /l)	15 (13–20)	17 (14–19)	15 (12–17)	21 (18-23)
Median (IQR)				
ALT (normal range ≤ 40 U /l)	11 (9–14)	10 (9–12)	9 (8–22)	14 (10–18)
Median (IQR)				
Platelet count	231 (197–260)	230 (171–251)	192 (171–222)	194 (187–216)
Median (IQR)				

## Data Availability

Data and methods used in the research are presented in detail in this article. All data relevant to the study are included in the article.
